# Genetic Determinants of Thrombin Generation and Their Relation to Venous Thrombosis: Results from the GAIT-2 Project

**DOI:** 10.1371/journal.pone.0146922

**Published:** 2016-01-19

**Authors:** Laura Martin-Fernandez, Andrey Ziyatdinov, Marina Carrasco, Juan Antonio Millon, Angel Martinez-Perez, Noelia Vilalta, Helena Brunel, Montserrat Font, Anders Hamsten, Juan Carlos Souto, José Manuel Soria

**Affiliations:** 1 Unit of Genomics of Complex Diseases, Biomedical Research Institute Sant Pau (IIB-Sant Pau), Barcelona, Spain; 2 Unit of Hemostasis and Thrombosis, Department of Hematology, IIB-Sant Pau, Hospital de la Santa Creu i Sant Pau, Barcelona, Spain; 3 Cardiovascular Genetics and Genomics Group, Atherosclerosis Research Unit, Department of Medicine, Karolinska Institutet, Karolinska University Hospital Solna, Stockholm, Sweden; Ottawa Hospital Research Institute, CANADA

## Abstract

**Background:**

Venous thromboembolism (VTE) is a common disease where known genetic risk factors explain only a small portion of the genetic variance. Then, the analysis of intermediate phenotypes, such as thrombin generation assay, can be used to identify novel genetic risk factors that contribute to VTE.

**Objectives:**

To investigate the genetic basis of distinct quantitative phenotypes of thrombin generation and its relationship to the risk of VTE.

**Patients/Methods:**

Lag time, thrombin peak and endogenous thrombin potential (ETP) were measured in the families of the Genetic Analysis of Idiopathic Thrombophilia 2 (GAIT-2) Project. This sample consisted of 935 individuals in 35 extended families selected through a proband with idiopathic thrombophilia. We performed also genome wide association studies (GWAS) with thrombin generation phenotypes.

**Results:**

The results showed that 67% of the variation in the risk of VTE is attributable to genetic factors. The heritabilities of lag time, thrombin peak and ETP were 49%, 54% and 52%, respectively. More importantly, we demonstrated also the existence of positive genetic correlations between thrombin peak or ETP and the risk of VTE. Moreover, the major genetic determinant of thrombin generation was the *F2* gene. However, other suggestive signals were observed.

**Conclusions:**

The thrombin generation phenotypes are strongly genetically determined. The thrombin peak and ETP are significantly genetically correlated with the risk of VTE. In addition, *F2* was identified as a major determinant of thrombin generation. We reported suggestive signals that might increase our knowledge to explain the variability of this important phenotype. Validation and functional studies are required to confirm GWAS results.

## Introduction

Venous thromboembolism (VTE) is a common disease involving genetic and environmental risk factors and their interactions [1]. VTE, which consist of deep vein thrombosis and pulmonary embolism, has an annual incidence of approximately 1 in 1000 individuals in developed countries [2]. Previous studies have estimated that the risk of VTE has a heritability of approximately 60% [3,4]. This means that the proportion of the variance that is attributable to genetic effects is approximately 60%. Specifically, hemostasis phenotypes have been used as intermediate phenotypes to identify novel genetic risk factors that contribute to thrombotic disease [3,5,6].

One of these intermediate phenotypes could be a measure of the thrombin generation assay, which measures the capacity to generate thrombin, a key enzyme in the coagulation cascade [7]. This phenotype is a global coagulation assay developed by Hemker *et al*. [8]. There are distinct quantitative phenotypes such as lag time, thrombin peak and the endogenous thrombin potential (ETP) representative of the dynamics of thrombin generation. Lag time corresponds to the time from the beginning of the test to the time when thrombin formation begins. Thrombin peak is defined as the highest thrombin concentration detectable. The ETP is determined from the area under the thrombin generation curve that measures the enzymatic capacity of thrombin generated during its lifetime [8,9].

Several studies have reported an association between these quantitative thrombin generation phenotypes and the risk of cardiovascular diseases using different test conditions. Specifically, an association has been described between thrombin generation and the risk of first VTE [10–13]. However, the results of the risk of recurrence are equivocal [11,14–16]. Thrombin generation phenotypes have been associated also with the risk of ischemic stroke [17,18] and acute myocardial infarction [19]. Also, the heritability of thrombin generation has been estimated previously at 32% [12]. These results indicate that the study of the genetic basis of thrombin generation phenotypes should identify novel genetic factors involved in the risk of VTE.

In addition to the Genetic Analysis of Idiopathic Thrombophilia 1 (GAIT-1) Project [3,20], we initiated another project—the Genetic Analysis of Idiopathic Thrombophilia 2 (GAIT-2) Project, using a new set of families. Several intermediate phenotypes were analyzed in the families of the GAIT-2 Project, including thrombin generation phenotypes. One aim of the present study was to determine the heritability of the risk of VTE as compared to the heritability values found in the GAIT-1 Project, and to estimate the genetic basis of 3 phenotypes related to thrombin generation. To evaluate their function as intermediate phenotypes with the risk of VTE, another aim was to estimate the phenotypic, genetic and environmental correlations of these 3 phenotypes with the risk of VTE. Finally, we wanted to perform genome wide association studies (GWAS) to identify susceptibility loci for thrombin generation phenotypes.

## Material and Methods

### Subjects

The Spanish families in our study were recruited in the GAIT-2 Project at the Hospital de la Santa Creu i Sant Pau of Barcelona, Spain.

The recruitment and the criteria used for inclusion were the same as that in GAIT-1 and have been described in detail previously [20]. Briefly, to be included in this study, a family was required to have at least 10 living individuals in 3 or more generations. Families were selected through a proband with idiopathic thrombophilia, which was defined as recurrent thrombotic events (at least one of which was spontaneous), a single spontaneous thrombotic episode plus a first-degree relative also affected, or onset of thrombosis before age 45. Thrombosis in these probands was considered idiopathic following the same criteria as in the GAIT-1 Project (excluding biological causes as antithrombin deficiency, Protein S and C deficiencies, activated protein C resistance, plasminogen deficiency, heparin cofactor II deficiency, Factor V Leiden, dysfibrogenemia, lupus anticoagulant and antiphospholipid antibodies). The subjects were interviewed by a physician to determine their health and reproductive history, current medications, alcohol consumption, use of sex hormones (oral contraceptives or hormonal replacement therapy) and their smoking history. Physical activity was also determined using the short form of the International Physical Activity Questionnaire (IPAQ) [21].

Our subjects were questioned also about previous episodes of venous and arterial thrombosis, the age at which these events occurred, and the presence of potentially correlated disorders such as diabetes, lipid disease, asthma, allergic rhinitis, atopic dermatitis, autoimmune disease and cancer. The residence of each subject was determined to assess the contribution of shared environmental influences (such as diet) common to members of the same household. The study was performed according to the Declaration of Helsinki. All procedures were reviewed and approved by the Institutional Review Board of the Hospital de la Santa Creu i Sant Pau, Barcelona, Spain. Adult subjects gave written informed consent for themselves and for their minor children.

This sample consisted of 935 individuals in 35 extended families. All of the pedigrees contained at least 3 generations, and 14 families had more than 3 generations. The individuals ranged in age from 2.6 to 101 years old (SD = 21.4), with a median age of 39.5 and approximately an equal number of males (465) and females (470). The depth and complexity of the pedigrees are illustrated in Table 1 with the number of pairs of relatives contained therein.

**Table 1:** Relative pairs.

n pairs	Kinship coefficient x 2	Relation
935	1	Self
1001	0.5	Parent-offspring
597	0.5	Siblings
467	0.25	Grandparent-grandchild
1248	0.25	Avuncular
7	0.25	Half siblings
3	0.25	Double 1st cousins
1807	0.125	3rd degree
1697	0.0625	4th degree
1296	0.03125	5th degree
349	0.015625	6th degree
177	0.0078125	7th degree
7116	0	Unrelated

The sample had 120 subjects with thromboembolism, including venous and arterial thrombosis. Specifically, they were 86 subjects with venous thrombosis, 47 with arterial thrombosis and 13 with both venous and arterial thrombosis. Of the 935 subjects, we obtained the thrombin generation phenotypes of 919 subjects.

### Blood Collection

Blood was collected by venipuncture following a 12-hour fast. Samples were collected in 1/10 volume containing 0.129 mol/L sodium citrate. None of the participants was using oral anticoagulants or heparins at the time of blood collection. Platelet-poor plasma (PPP) was obtained by centrifugation at 2000 *g* for 20 minutes at room temperature (22±2°C) and stored at −80°C before performing the thrombin generation assay. DNA was extracted from whole blood samples using a standard salting out procedure [22].

### Thrombin Generation Assay

Thrombin generation was evaluated in PPP according to the method described by Hemker *et al*. [23] by means of Fluoroskan Ascent (Thermo Labsystems, Helsinki, Finland), an automated fluorometer with a 390/460 nm filter set. Measurements were conducted on 80 μL of PPP and 20 μL of PPP reagent was added (Thrombinoscope BV, Maastricht, The Netherlands) consisting of a final concentration of 5 pM tissue factor and 4 μM phospholipids. The thrombin calibrator and the fluorogenic substrate with CaCl_2_ FluCa-Kit from Thrombinoscope BV (Maastricht, The Netherlands) were used. Thrombin generation curves were calculated using the Thrombinoscope^™^ software (Synapse BV, Maastricht, The Netherlands) and the parameters analyzed were lag time (min), thrombin peak (nM), and ETP (nM*min). The medians of crude lag time, crude thrombin peak and crude ETP were 2.67 min (first quartile 2.33 min and third quartile 3.08 min), 315.80 nM (first quartile 224.80 nM and third quartile 391.50 nM) and 1,594 nM*min (first quartile 1,260 nM*min and third quartile 1,980 nM*min). The intra-assay and inter-assay coefficients of variation for thrombin generation parameters were below 6% and below 8%, respectively.

### Genotyping and Imputation

We genotyped the samples with a combination of HumanOmniExpressExome-8v1.2 (324 individuals) and HumanCoreExome-12v1.1 (610 individuals). We applied inclusion filters in the datasets based on call rate (>98%), HWE (p-value >1.00x10^-06^) and MAF (>1%). We merged the data and obtained 395,556 SNPs in all of the samples. Then, we estimated haplotypes using SHAPEIT v2 [24] and imputed genotypes to the 1000 genomes phase 1 panel using IMPUTE2 [25]. We obtained 37,985,264 SNPs.

### Statistical Analyses

Prior to the data analyses, statistics logarithmic transformations were performed to normalize distributions in traits.

The statistical methods used in our study have been described elsewhere [3,26]. The analysis of heritability (*h*^*2*^, the relative proportion of phenotypic variance of the trait attributable to the additive effects of genes) was performed using the variance component method [27]. The total phenotypic variance was partitioned into three components: (1) an additive genetic variance that is caused by the sum of the average effects of all of the genes that influence the trait; (2) a shared environmental variance that is caused by the environmental factors that are common to members of a household (*c*^*2*^); and (3) a residual random environmental variance that is specific to each individual. The random environmental variance also includes non-additive genetic effects such as interactions between alleles within loci (dominance effects), interactions between alleles at different loci (epistatic effects), and effects caused by gene-environment interactions. Therefore, this model generally underestimates the role of genetics in the determination of the trait.

The covariances among individuals within a family that are due to additive genetic effects were estimated as a function of their expected genetic kinship relationships. Covariances among individuals that are due to shared environments were modeled by using the information whether individuals live in the same household. The power of this variance component approach of partition genetic and environmental effects stems from the high information content in the case of the extended pedigrees where families cut across multiple household [28]. Because the pedigrees were ascertained through a thrombophilic proband, all analyses included an ascertainment correction to allow unbiased estimation of parameters relevant to the general population [29,30].

Trait-specific covariates in the variance component models were evaluated from the following list of candidate covariates: age, sex, physical activity, oral contraceptives and smoking. The regression coefficient for the continuous covariate age represents the effect associated with a 1-year deviation from the mean age. The covariate age^2^ captured the non-linear relationship between the trait and age. The regression coefficients for the discrete covariates (female sex, physical activity, oral contraceptives and smoking) represent the effect of the covariate versus its absence. P-values of less than 0.05 were considered statistically significant.

The disease status of VTE is recorded as a binary trait encoding affected or unaffected status. In the statistical model, it is transformed to a continuous trait referred to as liability or susceptibility (risk) to the disease by means of probit link function. An interpretation of the effect of a continuous covariate like age in our model with probit link function follows a rule: the negative sign of the coefficient, for example for age covariate, means positive effect in the model (increase liability to the disease).

The correlations between a given pair of traits were analyzed by multivariate variance component models, which are an extension of the univariate model [27]. Similarly to the univariate model for estimation of the heritability of a single trait, the bivariate model partitioned the phenotypic covariances between traits into genetic and environmental components. The derived parameter of genetic correlation coefficient quantifies the pleiotropic genetic effects (i.e., one gene may have effects on several traits). This partition is potentially valuable, since hidden relationships between traits can be revealed [31]. By studying these traits in extended families, we can estimate robustly both the genetic (ρ_g_), and the environmental (ρ_e_) correlations between traits. The phenotypic correlation (ρ_p_) can be derived from these two constituent correlations and the heritabilities of the traits as follows:

$$\rho_{\text{p}}=\surd (h^{2}{}_{1}h^{2}{}_{2})\;\rho_{\text{g}}+\surd (1–h^{2}{}_{1})\;\surd (1-h^{2}{}_{2})\;\rho_{\text{e}}.$$

where *h*^2^_*1*_ and *h*^2^_*2*_ are the heritabilities for trait one and trait two.

For the association analysis with the imputed genotypes it was applied two excluding filters based on imputation score (info <0.3) and MAF (<1%). The final number of SNPs was 9,303,497. Analyses were performed using the measured genotype method by testing for genotype-specific differences in the means of traits while allowing for the nonindependence among family members. Genome wide significance was defined by p-values <5x10^−8^, and suggestive significance was defined by p-values <1x10^−5^. The association analysis was adjusted for *F2* G20210A mutation as known genetic determinant of thrombin generation, if needed.

All statistical analyses were performed employing the computer package Sequential Oligogenic Linkage Analysis Routines (SOLAR, version 8, official) [28]. SOLAR employs the maximum likelihood approach for variance component models with the standard likelihood ratio tests (LRT) to evaluate the statistical significance of the model parameters [32].

## Results

### Effect of covariates and heritabilities

The effects of significant covariates of the risk of VTE and thrombin generation phenotypes are shown in Table 2. The covariate age was significantly and positively related to the risk of VTE as well as to the lag time, thrombin peak and ETP. Furthermore, the results of the analysis of the covariate sex showed that women, in comparison with men, have significantly shorter lag times and greater thrombin peak values. Finally, use of oral contraceptives were related to a decreased lag time and associated with an increased thrombin peak and ETP. In contrast, smoking and physical activity were not related to the risk of VTE nor to thrombin generation phenotypes. Interestingly, significant covariates explained only from 4% to 10% of the variability of these traits. Residual kurtosis was within normal range.

**Table 2:** Significant covariates affecting the risk of VTE and thrombin generation.

Trait	Mean	SE (Mean)	Covariate	β	SE (β)	p-value	Variance due to covariates
VTE	NA	NA	AGE	-0.023*	0.004	6.28x10^-12^	NA
LT	1.02	0.01	AGE	0.001	0.0003	1.39x10^-03^	0.04
			SEX	-0.05	0.01	7.77x10^-04^	
			OC	-0.11	0.04	2.04x10^-03^	
TP	32.09	0.06	AGE	0.01	0.001	1.67x10^-06^	0.06
			AGE^2	-0.0002	0.00005	3.72x10^-05^	
			SEX	0.13	0.05	1.46x10^-02^	
			OC	0.72	0.13	8.16x10^-08^	
ETP	48.53	0.06	AGE	0.01	0.001	1.70x10^-18^	0.10
			AGE^2	-0.0003	0.00005	7.53x10^-10^	
			OC	0.87	0.14	9.81x10^-10^	

Mean and SE (Mean) indicate mean of the phenotype value and standard error; β, SE (β) and p-value: regression coefficient, standard error and p-value of regression coefficient; VTE: venous thromboembolism; NA: not applicable; OC: oral contraceptives; LT: Lag Time; TP: Thrombin Peak; and ETP: endogenous thrombin potential.

* The effect of AGE covariate to the risk of VTE is positive, as the negative sign of the coefficient means positive effect in this model, where the VTE response (binary) variable was transformed by probit link function.

The analysis of the contribution of genetics to the variability of each trait (trait heritability = *h*^*2*^) was performed after the correction by its significant covariates. It is notable that genetic factors accounted for 67% of the variation in the risk of VTE. Furthermore, the heritabilities of lag time, thrombin peak and ETP were estimated as 49%, 54% and 52%, respectively. The estimates of additive genetic effects and the household effect on the variability of the traits are shown in Table 3.

**Table 3:** Heritabilities and household effect.

Trait	h^2^	SE (h^2^)	p-value (h^2^)	c^2^	SE (c^2^)	p-value (c^2^)
VTE	0.67	0.17	1.60x10^-06^	-*	-	-
LT	0.49	0.07	3.32x10^-15^	0.21	0.05	2.80x10^-06^
TP	0.54	0.07	3.14x10^-16^	0.27	0.05	9.66x10^-10^
ETP	0.52	0.06	5.71x10^-18^	0.23	0.05	2.27x10^-08^

h^2^, SE (h^2^) and p-value (h^2^) indicate heritability, standard error and p-value of the heritability; c^2^, SE (c^2^) and p-value (c^2^): household effect, standard error and p-value of the household effect; VTE: venous thromboembolism; LT: Lag Time; TP: Thrombin Peak; and ETP: endogenous thrombin potential. The covariates used in each model were reported in Table 2.

*The household effect (c^2^) was removed from the model of VTE trait, as its estimation was 0.

### Phenotypic, genetic and environmental correlations of thrombin generation phenotypes with the risk of VTE

The lag time was not phenotypically correlated with VTE. In contrast, the thrombin peak and ETP showed significant and positive phenotypic correlations with VTE (Table 4).

**Table 4:** Phenotypic, genetic and environmental correlations of thrombin generation phenotypes with the risk of VTE.

Trait 1	Trait 2	ρ_p_	p-value (ρ_p_)	ρ_g_	SE (ρ_g_)	p-value (ρ_g_)	ρ_e_	SE (ρ_e_)	p-value (ρ_e_)
VTE	LT	0.05	3.96x10^-01^	0.21	0.22	3.53x10^-01^	-0.04	0.13	7.50x10^-01^
VTE	TP	0.16	1.10x10^-02^	0.47	0.25	3.21x10^-02^	-0.05	0.15	7.25x10^-01^
VTE	ETP	0.20	1.11x10^-03^	0.50	0.28	3.31x10^-02^	0.04	0.14	7.96x10^-01^

ρ_p_ and p-value (ρ_p_) indicate phenotypic correlation and p-value of phenotypic correlation; ρ_g_, SE (ρ_g_) and p-value (ρ_g_): genetic correlation, standard error and p-value of genetic correlation; ρ_e_, SE (ρ_e_) and p-value (ρ_e_): environmental correlation, standard error and p-value of environmental correlation; VTE: venous thromboembolism; LT: Lag Time; TP: Thrombin Peak; and ETP: endogenous thrombin potential.

The lag time did not show significant genetic or environmental correlations. In contrast, and most relevant, the thrombin peak and the ETP showed significant genetic correlations with the risk of VTE, but we did not find environmental correlations (Table 4).

### Genetic correlations among thrombin generation phenotypes

The lag time showed significant and negative genetic correlations with the thrombin peak and ETP, but they were not correlated environmentally. Otherwise, the significant genetic and environmental correlations between the thrombin peak and ETP were positive (Table 5).

**Table 5:** Genetic and environmental correlations among thrombin generation phenotypes.

Trait 1	Trait 2	ρ_p_	p-value (ρ_p_)	ρ_g_	SE (ρ_g_)	p-value (ρ_g_)	ρ_e_	SE (ρ_e_)	p-value (ρ_e_)
TP	LT	-0.40	3.39x10^-28^	-0.50	0.06	2.21x10^-11^	-0.19	0.10	6.74x10^-02^
ETP	LT	-0.14	2.10x10^-04^	-0.23	0.08	5.38x10^-03^	0.02	0.09	8.07x10^-01^
ETP	TP	0.85	3.23x10^-48^	0.87	0.02	1.52x10^-37^	0.82	0.03	2.14x10^-12^

ρ_p_ and p-value (ρ_p_) indicate phenotypic correlation and p-value of phenotypic correlation; ρ_g_, SE (ρ_g_) and p-value (ρ_g_): genetic correlation, standard error and p-value of genetic correlation; ρ_e_, SE (ρ_e_) and p-value (ρ_e_): environmental correlation, standard error and p-value of environmental correlation; LT: Lag Time; TP: Thrombin Peak; and ETP: endogenous thrombin potential.

### GWAS

**Fig 1:**
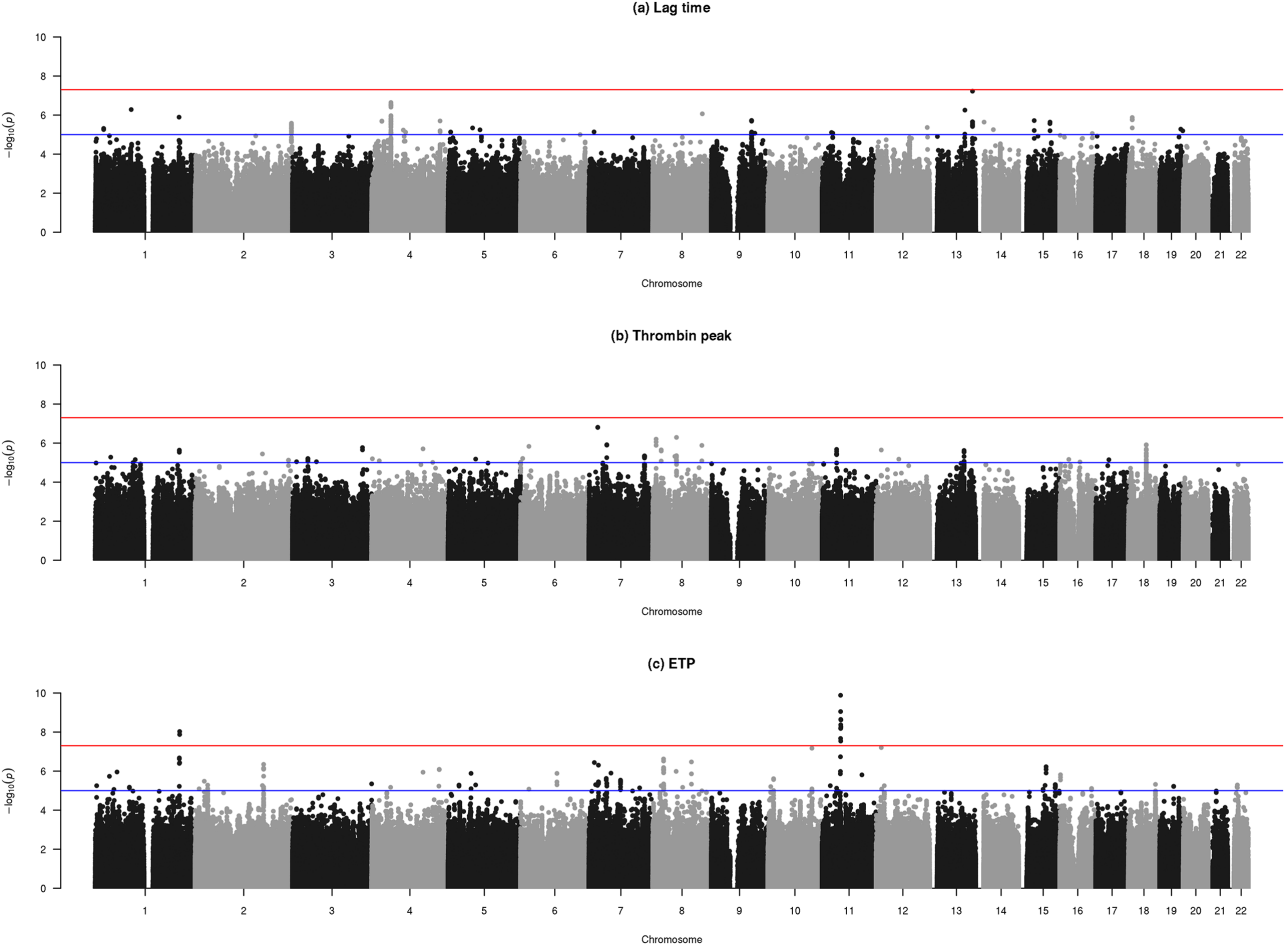
Manhattan plots of the genome wide association studies on the 3 thrombin generation phenotypes. Lag time (a), thrombin peak (b), and ETP (c). Dots correspond to SNPs organized by chromosomal order and position and the *y* axis shows the statistical significance expressed as -log_10_ of the p-values. The horizontal lines correspond to genome wide significant threshold taken at 5×10^−8^ and genome wide suggestive significance threshold at 1x10^−5^.

No inflation of the test statistic was observed (λ = 1.05, λ = 1.03, λ = 1 and λ = 0.99 for the risk of VTE, lag time, thrombin peak and ETP, respectively). No variants showed genome wide significance for association with the risk of VTE (data not shown). Manhattan plots of the results from genome wide associations on the thrombin generation traits are shown in Fig 1. Briefly, no variant attained genome wide significance for association with lag time or thrombin peak. In contrast, 2 peaks on Chromosomes 1 and 11 reached genome wide significance level (p-value = 5x10^-08^) in association with the ETP trait. The significant SNPs rs61828128 (MAF = 2.18% and p-value = 1.33x10^-08^) and rs61828133 (MAF = 2.18% and p-value = 9.36x10^-09^) on Chromosome 1 are located in the *HHAT* gene. Interestingly, the SNP rs1799963 (MAF = 2.82% and p-value = 2.25x10^-09^) in *F2* gene is included in the association peak at chromosome 11 region. After adjustment for G20210A (rs1799963) mutation in the *F2* gene, the signals did not remain significant. However, our results suggested associations involved in both thrombin peak and ETP traits in or near *IRF6* (rs75594643; MAF = 4.31%, p-value = 6.13x10^-06^ for thrombin peak, p-value = 2.94x10^-06^ for ETP, and rs1474608; MAF = 3.80%, p-value = 4.66x10^-06^ for thrombin peak, p-value = 2.46x10^-06^ for ETP), *OCLN* (rs76696742; MAF = 1.13%, p-value = 5.19x10^-06^ for thrombin peak, p-value = 6.59x10^-06^ for ETP), *CDKAL1* (rs16884308; MAF = 1.16%, p-value = 2.57x10^-06^ for thrombin peak, p-value = 1.47x10^-06^ for ETP), *CAMK2B* (rs180694332; MAF = 1.72%, p-value = 6.55x10^-06^ for thrombin peak, p-value = 5.58x10^-06^ for ETP), and *NUDCD3* (rs144256107; MAF = 1.99%, p-value = 9.80x10^-06^ for thrombin peak, p-value = 1.26x10^-06^ for ETP) genes. We defined as “near” a distance between 1 and 1,500 bp upstream.

## Discussion

To investigate the risk of diseases, the study of complex intermediate phenotypes provide a useful means to identify genetic risk factors. The intermediate phenotypes are closer to the action of genes than the presence or absence of a complex disease. In addition, the susceptibility to a complex disease is primarily a process that represents an unobservable continuous liability. This means that this variable can not be measured directly in an individual, and consequently the use of intermediate phenotypes is statistically more powerful [33]. Our study demonstrates that the thrombin generation assay is a useful tool for the study of the risk of VTE.

Using a variance component method and maximum likelihood estimations age was found to be the covariate with statistically significant effect on the risk of VTE. This is not surprising since the covariate age has been associated previously with VTE [1]. Except for smoking and physical activity, all covariates, including age, sex and oral contraceptives, were significantly related to the parameters of thrombin generation assay. Specifically, age, sex and oral contraceptives influenced the lag time. The thrombin peak was significantly related with age, age^2^, sex and oral contraceptives and the ETP was influenced by age, age^2^ and oral contraceptives. These results are consistent with previous reports under different experimental conditions. Specifically, it has been reported that thrombin generation increases with age and is higher in women than in men [34]. In addition, oral contraceptives have been related also with an increased thrombin generation [11,35].

Our study was based on the recruitment of families, which provided direct data of familial transmission and allowed an estimation of genetic factors affecting the variation in the risk of VTE within a Spanish population [3]. It is notable that, in our population, genetic factors accounted for 67% of the variation in the risk of VTE. This high heritability is similar to what we reported previously in the GAIT-1 Project study [3]. The heritabilities of thrombin generation phenotypes in this new set of families ranged from 49% to 54%. These findings are consistent with previous published data [12]. These high heritabilities indicate that genes play a major role in the determination of the variability of these parameters.

One of our most important results is that they add to the previous evidence of the relationship between the risk of VTE and thrombin generation. For the first time to our knowledge, we determined genetic correlations between the susceptibility to VTE and thrombin peak or ETP. Not surprisingly, we did not find phenotypic, genetic nor environmental correlations between the risk of VTE and lag time. Our results agree with previous publications which reported no association between the lag time and the risk of first or recurrent VTE [11]. In contrast, thrombin peak and ETP showed positive phenotypic correlations with the risk of VTE which are similar to previous evidence. Interestingly, our study is the first that shows strong evidence that the positive associations between the risk of VTE and thrombin peak or ETP is caused by pleiotropic factors. These results provide evidence that there are genes acting jointly on both the risk of VTE and thrombin peak or ETP. Therefore, we believe that it is prudent to use the thrombin generation assay as an intermediate phenotype to identify novel genes that affect the risk of VTE.

Taking together, our results support the hypothesis that the thrombin generation test is a very useful test to investigate the risk of VTE. Specifically, thrombin generation phenotypes have been associated significantly with genetic variants in haemostatic genes such as *F5*, *F2*, *FGA*, *F10*, *F12* and *TFPI* [7,36]. It has been reported recently that there is a new association between thrombin generation variability and the *ORM1* locus [37]. It is important to note that known genetic risk factors have been estimated to account for <30% of VTE cases [38]. Consequently, this global coagulation assay might identify novel genetic variants that contribute to the heritability of the risk of VTE.

In agreement with a previous study [37] we observed a high significant association between thrombin generation and prothrombin G20210A mutation. This genetic variation is a well-known genetic risk factor for VTE [1]. In addition, we observed suggestive signals (presented in at least two out of the three phenotypes studied) that might increase our knowledge to explain the variability of thrombin generation. However, we do not have biological evidences to relate these observed signals with the phenotypes due to the lack of information about functionality in the data bases. Thus, further studies are needed to elucidate the implication of these new genes with thrombin generation and the risk of VTE.

The thrombin generation assay is sensitive to preanalytic conditions and there is no standardized protocol [39,40]. In detail, we used 5 pM tissue factor as trigger in PPP. At this concentration, thrombin generation assay is sensitive to factors VIII and IX. Lower concentrations (1 pM) of tissue factor could lead to an increase of the variability of the assay. It is difficult to compare various studies considering that this assay can be performed under widely different laboratory conditions [41]. Despite this, our results are consistent with those of previous studies, but add important new data.

In summary, the high heritabilities that we found indicate that genetic factors play a significant role in the risk of VTE and in the thrombin generation test in this population. In addition, the significant genetic correlations suggest that there are pleiotropic genetic effects between the risk of VTE and thrombin peak or ETP. Finally, our study indicates that the use of the thrombin generation assay should help to detect genes responsible for thrombophilia. In fact, from results of the GWAS, *F2* has been shown as the main contributor to thrombin generation.
